# Phyllo-poly[[μ_2_-1,4-bis­(cyclo­hexyl­sulfanylmeth­yl)benzene-κ^2^ *S*:*S*′](μ_2_-nitrato-κ^2^ *O*:*O*′)silver(I)]

**DOI:** 10.1107/S1600536809007910

**Published:** 2009-03-11

**Authors:** Tae Ho Kim, Yong Woon Shin, Ki-Min Park, Jineun Kim

**Affiliations:** a Department of Chemistry, Gyeongsang National University, Jinju 660-701, Republic of Korea; b Test & Analytical Laboratory, Korea Food & Drug Administration, 123-7 Yongdang-dong, Busan 608-829, Republic of Korea; c Research Institute of Natural Sciences, Gyeongsang National University, Jinju 660-701, Republic of Korea; d Department of Chemistry, Gyeongsang National University, Jinju 660-701, Republic of Korea

## Abstract

The title compound, [Ag(NO_3_)(C_20_H_30_S_2_)]_*n*_, was synthesized by the reaction of silver nitrate and 1,4-bis­(cyclo­hexyl­thio­meth­yl)benzene (bctmb) in acetonitrile. The coordination polymer exhibits a two-dimensional layer structure. The layers are wave-like and parallel to the crystallographic *ac* plane; Ag^I^ ions are linked by the bctmb ligands and nitrate anions along the crystallographic *a* and *c* directions, respectively. In addition, the crystal structure is stabilized by C—H⋯O hydrogen bonds.

## Related literature

For the synthesis of the ligand, see: Kim *et al.* (2008 ▶). For related structures, see: Kim *et al.* (2007 ▶). For structures with Ni(II) in trigonal-pyramidal coordination, see: Cho *et al.* (2007 ▶). For potential applications of coordination polymers, see: Young & Hanton (2008 ▶).
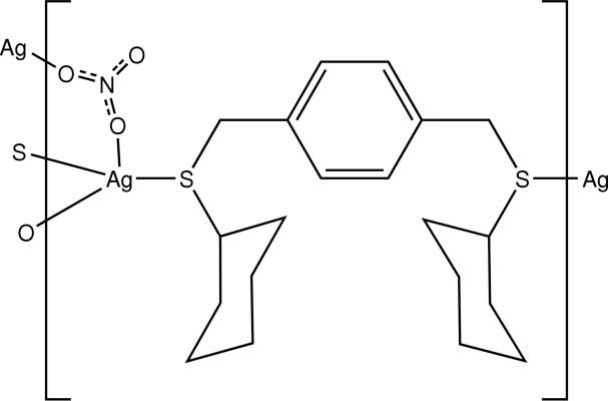


## Experimental

### 

#### Crystal data

[Ag(NO_3_)(C_20_H_30_S_2_)]
*M* *_r_* = 504.44
Monoclinic, 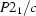

*a* = 12.1053 (6) Å
*b* = 20.719 (1) Å
*c* = 8.5973 (4) Å
β = 92.256 (1)°
*V* = 2154.61 (18) Å^3^
*Z* = 4
Mo *K*α radiation
μ = 1.15 mm^−1^
*T* = 173 K
0.30 × 0.20 × 0.10 mm

#### Data collection

Bruker SMART CCD area-detector diffractometer
Absorption correction: multi-scan (*SADABS*; Sheldrick, 1996 ▶) *T*_min_ = 0.724, *T*_max_ = 0.894
13362 measured reflections
4804 independent reflections
3174 reflections with *I* > 2σ(*I*)
*R*_int_ = 0.042

#### Refinement

*R*[*F* ^2^ > 2σ(*F* ^2^)] = 0.046
*wR*(*F* ^2^) = 0.115
*S* = 1.04
4804 reflections
238 parameters
H-atom parameters constrained
Δρ_max_ = 0.90 e Å^−3^
Δρ_min_ = −1.00 e Å^−3^

### 

Data collection: *SMART* (Bruker, 2000 ▶); cell refinement: *SAINT-Plus* (Bruker, 2000 ▶); data reduction: *SAINT-Plus*; program(s) used to solve structure: *SHELXTL* (Sheldrick, 2008 ▶); program(s) used to refine structure: *SHELXTL*; molecular graphics: *SHELXTL*; software used to prepare material for publication: *SHELXTL*.

## Supplementary Material

Crystal structure: contains datablocks global, I. DOI: 10.1107/S1600536809007910/lx2093sup1.cif

Structure factors: contains datablocks I. DOI: 10.1107/S1600536809007910/lx2093Isup2.hkl

Additional supplementary materials:  crystallographic information; 3D view; checkCIF report

## Figures and Tables

**Table 1:** Hydrogen-bond geometry (Å, °)

*D*—H⋯*A*	*D*—H	H⋯*A*	*D*⋯*A*	*D*—H⋯*A*
C14—H14*A*⋯O3^i^	0.99	2.60	3.416 (7)	140
C14—H14*B*⋯O3^ii^	0.99	2.47	3.199 (6)	130
C7—H7*B*⋯O2^iii^	0.99	2.43	3.118 (6)	126

Symmetry codes: (i) 
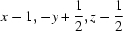
; (ii) 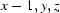; (iii) 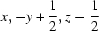.

**Fig. 1.**
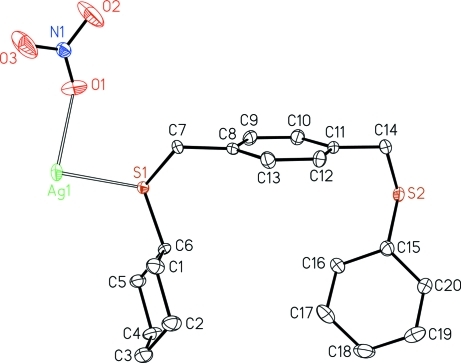
The molecular structure of the title compound, showing displacement ellipsoids drawn at the 30% probability level. All H atoms have been omitted for clarity.

**Fig. 2.**
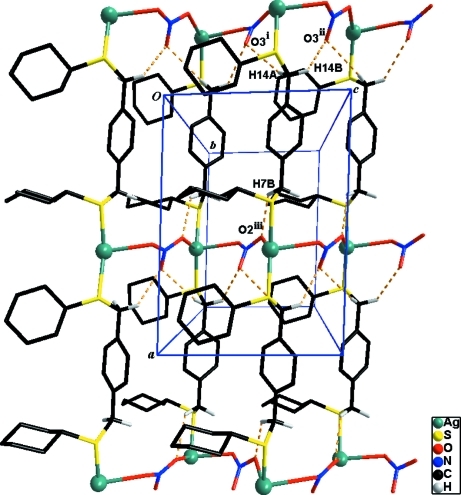
Two-dimensional network structure showing C—H···O interactions. All H atoms except those relating C—H···O interactions have been omitted for clarity. [Symmetry codes: (i) x-1, -y+1/2, z-1/2; (ii) x-1, y, z; (iii) x, -y+1/2, z-1/2.]

## supplementary crystallographic information

### Comment

An increasing interest has been directed toward the study of new coordination polymers owing to potential applications (Young & Hanton, 2008). The scant research on the coordination polymers with dithioether ligands prompted us to investigate the possibility of diverse structures. Therefore, we designed and synthesized 1,4-bis(cyclohexylthiomethyl)benzene (bctmb) as a dithioether ligand. Synthesis of the bctmb ligand has been published previously (Kim, *et al.*, 2008).

The title compound, phyllo-poly[(µ_2_-nitrato-κ^2^*O*:*O*')(µ_2_-1,4-bis(cyclohexylthiomethyl)benzene- κ^2^*S*:*S*') silver(I)], [Ag(NO_3_)(C_20_H_30_S_2_)]*_n_* was synthesized by self-assembly of silver nitrate and the bctmb ligand in acetonitrile (Kim *et al.*, 2007) (Fig. 1). The coordination number of Ag is four and the Ag atom is a slightly distorted trigonal pyramidal geometry, in which an O atom (O2) from nitrate anion and two S atoms from two different bctmb ligands form a basal plane and an O atom (O1) from neighboring nitrate anion is occupied apical position. The Ag atom is slightly apart from this basal plane (0.123 (2) Å). Each Ag^I^ ions is linked by the bctmb ligands to form 1D chain along the *a* axis. These chains are connected by bidentate nitrate anions in a bridging mode to generate 2D layer structure, as shown in Fig. 2. The layers are wavy and parallel to the crystallographic *ac* plane. The packing structure is stabilized by C—H···O hydrogen bonds (Table 1 & Fig. 2).

### Experimental

The title compound was synthesized by self-assembly of stoichiometric amounts of silver nitrate and the bctmb ligands in acetonitrile (Kim *et al.*, 2007). Single crystals suitable for X-ray analysis were obtained by evaporation of a solution of the title compound in acetonitrile.

### Refinement

All H-atoms were positioned geometrically and refined using a riding model with d(C—H) = 0.93 Å, *U*_iso_ =1.2*U*_eq_(C) for aromatic and 0.97 Å, *U*_iso_ = 1.2*U*_eq_(C) for CH_2_ atoms.

### Crystal data

**Table:**

[Ag(NO_3_)(C_20_H_30_S_2_)]	*F*(000) = 1040
*M**_r_* = 504.44	*D*_x_ = 1.555 Mg m^−^^3^
Monoclinic, *P*2_1_/*c*	Mo *K*α radiation, λ = 0.71073 Å
Hall symbol: -P 2ybc	Cell parameters from 4031 reflections
*a* = 12.1053 (6) Å	θ = 2.6–27.6°
*b* = 20.719 (1) Å	µ = 1.15 mm^−^^1^
*c* = 8.5973 (4) Å	*T* = 173 K
β = 92.256 (1)°	Plate, colorless
*V* = 2154.61 (18) Å^3^	0.30 × 0.20 × 0.10 mm
*Z* = 4	

### Data collection

**Table:**

Bruker SMART CCD area-detector diffractometer	4804 independent reflections
Radiation source: fine-focus sealed tube	3174 reflections with *I* > 2σ(*I*)
graphite	*R*_int_ = 0.042
Detector resolution: 10.0 pixels mm^-1^	θ_max_ = 27.3°, θ_min_ = 1.7°
φ and ω scans	*h* = −12→15
Absorption correction: multi-scan (*SADABS*; Sheldrick, 1996)	*k* = −26→23
*T*_min_ = 0.724, *T*_max_ = 0.894	*l* = −11→9
13362 measured reflections	

### Refinement

**Table:**

Refinement on *F*^2^	Primary atom site location: structure-invariant direct methods
Least-squares matrix: full	Secondary atom site location: difference Fourier map
*R*[*F*^2^ > 2σ(*F*^2^)] = 0.046	Hydrogen site location: inferred from neighbouring sites
*wR*(*F*^2^) = 0.115	H-atom parameters constrained
*S* = 1.04	*w* = 1/[σ^2^(*F*_o_^2^) + (0.0558*P*)^2^ + 0.7474*P*] where *P* = (*F*_o_^2^ + 2*F*_c_^2^)/3
4804 reflections	(Δ/σ)_max_ = 0.001
238 parameters	Δρ_max_ = 0.90 e Å^−^^3^
0 restraints	Δρ_min_ = −1.00 e Å^−^^3^

### Special details

**Table:**

Geometry. All esds (except the esd in the dihedral angle between two l.s. planes) are estimated using the full covariance matrix. The cell esds are taken into account individually in the estimation of esds in distances, angles and torsion angles; correlations between esds in cell parameters are only used when they are defined by crystal symmetry. An approximate (isotropic) treatment of cell esds is used for estimating esds involving l.s. planes.
Refinement. Refinement of F^2^ against ALL reflections. The weighted R-factor wR and goodness of fit S are based on F^2^, conventional R-factors R are based on F, with F set to zero for negative F^2^. The threshold expression of F^2^ > 2sigma(F^2^) is used only for calculating R-factors(gt) etc. and is not relevant to the choice of reflections for refinement. R-factors based on F^2^ are statistically about twice as large as those based on F, and R- factors based on ALL data will be even larger.

### Fractional atomic coordinates and isotropic or equivalent isotropic displacement parameters (Å^2^)

**Table:**

	*x*	*y*	*z*	*U*_iso_*/*U*_eq_	
Ag1	0.60132 (3)	0.147912 (17)	0.59553 (4)	0.03340 (12)	
S1	0.40490 (8)	0.10911 (5)	0.59796 (11)	0.0245 (2)	
S2	−0.20353 (9)	0.11318 (6)	0.59401 (12)	0.0332 (3)	
O1	0.5747 (3)	0.16475 (16)	0.8814 (4)	0.0538 (10)	
O2	0.5492 (4)	0.2420 (2)	1.0367 (7)	0.1033 (19)	
O3	0.6861 (4)	0.2458 (2)	0.9036 (6)	0.1007 (19)	
N1	0.6032 (3)	0.2176 (2)	0.9376 (4)	0.0391 (7)	
C1	0.3798 (4)	0.1476 (2)	0.2840 (5)	0.0330 (10)	
H1A	0.4608	0.1544	0.2867	0.040*	
H1B	0.3442	0.1886	0.3135	0.040*	
C2	0.3396 (4)	0.1280 (2)	0.1194 (5)	0.0371 (11)	
H2A	0.2579	0.1249	0.1154	0.044*	
H2B	0.3608	0.1618	0.0449	0.044*	
C3	0.3879 (4)	0.0640 (2)	0.0714 (5)	0.0381 (11)	
H3A	0.4691	0.0682	0.0658	0.046*	
H3B	0.3577	0.0522	−0.0335	0.046*	
C4	0.3611 (4)	0.0111 (2)	0.1862 (5)	0.0376 (11)	
H4A	0.3976	−0.0295	0.1561	0.045*	
H4B	0.2802	0.0036	0.1834	0.045*	
C5	0.4004 (4)	0.0300 (2)	0.3512 (5)	0.0318 (10)	
H5A	0.4821	0.0330	0.3564	0.038*	
H5B	0.3784	−0.0040	0.4250	0.038*	
C6	0.3511 (3)	0.09466 (19)	0.3992 (4)	0.0236 (9)	
H6	0.2688	0.0903	0.4004	0.028*	
C7	0.3416 (3)	0.1852 (2)	0.6496 (5)	0.0250 (9)	
H7A	0.3716	0.1986	0.7534	0.030*	
H7B	0.3629	0.2185	0.5737	0.030*	
C8	0.2171 (3)	0.1830 (2)	0.6532 (4)	0.0242 (9)	
C9	0.1638 (4)	0.1424 (2)	0.7516 (5)	0.0341 (10)	
H9	0.2063	0.1134	0.8153	0.041*	
C10	0.0497 (4)	0.1424 (2)	0.7606 (5)	0.0346 (10)	
H10	0.0150	0.1137	0.8297	0.041*	
C11	−0.0144 (3)	0.1847 (2)	0.6682 (5)	0.0312 (10)	
C12	0.0387 (4)	0.2255 (2)	0.5700 (5)	0.0347 (11)	
H12	−0.0035	0.2547	0.5068	0.042*	
C13	0.1531 (3)	0.2248 (2)	0.5610 (5)	0.0303 (10)	
H13	0.1878	0.2531	0.4913	0.036*	
C14	−0.1381 (3)	0.1853 (2)	0.6764 (5)	0.0393 (12)	
H14A	−0.1676	0.2237	0.6200	0.047*	
H14B	−0.1579	0.1894	0.7867	0.047*	
C15	−0.1609 (4)	0.1157 (2)	0.3921 (5)	0.0313 (10)	
H15	−0.1399	0.1611	0.3665	0.038*	
C16	−0.0612 (4)	0.0726 (3)	0.3729 (5)	0.0459 (13)	
H16A	−0.0793	0.0282	0.4058	0.055*	
H16B	0.0009	0.0884	0.4410	0.055*	
C17	−0.0258 (4)	0.0716 (3)	0.2038 (6)	0.0602 (16)	
H17A	−0.0005	0.1151	0.1737	0.072*	
H17B	0.0365	0.0411	0.1935	0.072*	
C18	−0.1225 (4)	0.0509 (3)	0.0960 (5)	0.0504 (14)	
H18A	−0.0999	0.0524	−0.0133	0.060*	
H18B	−0.1433	0.0059	0.1200	0.060*	
C19	−0.2211 (4)	0.0948 (2)	0.1153 (5)	0.0391 (7)	
H19A	−0.2018	0.1392	0.0838	0.047*	
H19B	−0.2834	0.0798	0.0464	0.047*	
C20	−0.2563 (4)	0.0952 (3)	0.2821 (5)	0.0435 (12)	
H20A	−0.2815	0.0515	0.3110	0.052*	
H20B	−0.3191	0.1253	0.2924	0.052*	

### Atomic displacement parameters (Å^2^)

**Table:**

	*U*^11^	*U*^22^	*U*^33^	*U*^12^	*U*^13^	*U*^23^
Ag1	0.01609 (16)	0.0475 (2)	0.0365 (2)	0.00432 (16)	0.00031 (12)	−0.00270 (17)
S1	0.0197 (5)	0.0337 (6)	0.0199 (5)	0.0066 (4)	−0.0015 (4)	−0.0037 (4)
S2	0.0194 (5)	0.0523 (7)	0.0277 (6)	0.0080 (5)	−0.0005 (4)	−0.0018 (5)
O1	0.083 (3)	0.046 (2)	0.0318 (19)	−0.010 (2)	−0.0063 (18)	−0.0079 (16)
O2	0.092 (3)	0.066 (3)	0.159 (5)	−0.026 (3)	0.093 (4)	−0.053 (3)
O3	0.091 (4)	0.072 (3)	0.146 (5)	−0.035 (3)	0.089 (3)	−0.039 (3)
N1	0.0396 (18)	0.0507 (19)	0.0266 (15)	0.0039 (16)	−0.0033 (13)	−0.0020 (14)
C1	0.040 (3)	0.031 (2)	0.028 (2)	0.009 (2)	0.0021 (19)	0.0002 (19)
C2	0.047 (3)	0.040 (3)	0.024 (2)	0.008 (2)	−0.004 (2)	0.0021 (19)
C3	0.040 (3)	0.050 (3)	0.024 (2)	0.006 (2)	−0.0027 (19)	0.002 (2)
C4	0.051 (3)	0.039 (3)	0.022 (2)	0.004 (2)	−0.010 (2)	−0.0053 (19)
C5	0.037 (3)	0.035 (2)	0.023 (2)	0.008 (2)	−0.0049 (18)	−0.0047 (18)
C6	0.0213 (19)	0.035 (2)	0.0139 (18)	0.0029 (18)	−0.0040 (15)	−0.0016 (17)
C7	0.0176 (19)	0.032 (2)	0.026 (2)	0.0067 (18)	0.0010 (16)	−0.0058 (18)
C8	0.020 (2)	0.035 (2)	0.0176 (19)	0.0045 (18)	0.0006 (15)	−0.0057 (17)
C9	0.028 (2)	0.054 (3)	0.020 (2)	0.005 (2)	−0.0042 (17)	0.002 (2)
C10	0.025 (2)	0.056 (3)	0.022 (2)	0.002 (2)	0.0025 (17)	0.003 (2)
C11	0.018 (2)	0.053 (3)	0.023 (2)	0.006 (2)	−0.0013 (16)	−0.013 (2)
C12	0.028 (2)	0.041 (3)	0.034 (2)	0.014 (2)	−0.0082 (19)	−0.003 (2)
C13	0.028 (2)	0.036 (2)	0.027 (2)	0.007 (2)	0.0044 (18)	0.0048 (19)
C14	0.020 (2)	0.059 (3)	0.039 (3)	0.008 (2)	0.0005 (19)	−0.019 (2)
C15	0.027 (2)	0.040 (3)	0.028 (2)	0.001 (2)	0.0035 (18)	0.0017 (19)
C16	0.036 (3)	0.066 (4)	0.036 (3)	0.017 (3)	0.001 (2)	−0.012 (2)
C17	0.041 (3)	0.090 (4)	0.050 (3)	0.008 (3)	0.021 (3)	−0.016 (3)
C18	0.061 (4)	0.063 (3)	0.028 (3)	−0.007 (3)	0.011 (2)	−0.011 (2)
C19	0.0396 (18)	0.0507 (19)	0.0266 (15)	0.0039 (16)	−0.0033 (13)	−0.0020 (14)
C20	0.031 (3)	0.064 (3)	0.035 (3)	0.008 (2)	−0.004 (2)	−0.008 (2)

### Geometric parameters (Å, °)

**Table:**

Ag1—O2^i^	2.415 (4)	C7—H7B	0.9900
Ag1—S2^ii^	2.4699 (11)	C8—C9	1.371 (6)
Ag1—S1	2.5108 (11)	C8—C13	1.389 (5)
Ag1—O1	2.516 (3)	C9—C10	1.387 (6)
S1—C7	1.816 (4)	C9—H9	0.9500
S1—C6	1.829 (3)	C10—C11	1.397 (6)
S2—C14	1.822 (5)	C10—H10	0.9500
S2—C15	1.831 (4)	C11—C12	1.372 (6)
S2—Ag1^iii^	2.4699 (11)	C11—C14	1.503 (5)
O1—N1	1.241 (5)	C12—C13	1.390 (6)
O2—N1	1.205 (5)	C12—H12	0.9500
O2—Ag1^iv^	2.415 (4)	C13—H13	0.9500
O3—N1	1.207 (5)	C14—H14A	0.9900
C1—C6	1.527 (6)	C14—H14B	0.9900
C1—C2	1.532 (6)	C15—C16	1.515 (6)
C1—H1A	0.9900	C15—C20	1.524 (6)
C1—H1B	0.9900	C15—H15	1.0000
C2—C3	1.513 (6)	C16—C17	1.532 (7)
C2—H2A	0.9900	C16—H16A	0.9900
C2—H2B	0.9900	C16—H16B	0.9900
C3—C4	1.519 (6)	C17—C18	1.525 (7)
C3—H3A	0.9900	C17—H17A	0.9900
C3—H3B	0.9900	C17—H17B	0.9900
C4—C5	1.529 (5)	C18—C19	1.515 (7)
C4—H4A	0.9900	C18—H18A	0.9900
C4—H4B	0.9900	C18—H18B	0.9900
C5—C6	1.531 (6)	C19—C20	1.512 (6)
C5—H5A	0.9900	C19—H19A	0.9900
C5—H5B	0.9900	C19—H19B	0.9900
C6—H6	1.0000	C20—H20A	0.9900
C7—C8	1.510 (5)	C20—H20B	0.9900
C7—H7A	0.9900		
			
O2^i^—Ag1—S2^ii^	121.08 (12)	C9—C8—C7	121.7 (4)
O2^i^—Ag1—S1	93.69 (12)	C13—C8—C7	120.2 (4)
S2^ii^—Ag1—S1	144.39 (4)	C8—C9—C10	121.8 (4)
O2^i^—Ag1—O1	91.78 (15)	C8—C9—H9	119.1
S2^ii^—Ag1—O1	101.83 (10)	C10—C9—H9	119.1
S1—Ag1—O1	83.01 (9)	C9—C10—C11	120.0 (4)
C7—S1—C6	103.42 (18)	C9—C10—H10	120.0
C7—S1—Ag1	97.63 (13)	C11—C10—H10	120.0
C6—S1—Ag1	110.32 (14)	C12—C11—C10	118.3 (4)
C14—S2—C15	102.2 (2)	C12—C11—C14	121.0 (4)
C14—S2—Ag1^iii^	99.21 (14)	C10—C11—C14	120.7 (4)
C15—S2—Ag1^iii^	107.56 (14)	C11—C12—C13	121.3 (4)
N1—O1—Ag1	117.3 (3)	C11—C12—H12	119.4
N1—O2—Ag1^iv^	113.5 (3)	C13—C12—H12	119.4
O2—N1—O3	116.7 (4)	C8—C13—C12	120.6 (4)
O2—N1—O1	119.6 (5)	C8—C13—H13	119.7
O3—N1—O1	123.6 (5)	C12—C13—H13	119.7
C6—C1—C2	109.7 (3)	C11—C14—S2	113.2 (3)
C6—C1—H1A	109.7	C11—C14—H14A	108.9
C2—C1—H1A	109.7	S2—C14—H14A	108.9
C6—C1—H1B	109.7	C11—C14—H14B	108.9
C2—C1—H1B	109.7	S2—C14—H14B	108.9
H1A—C1—H1B	108.2	H14A—C14—H14B	107.8
C3—C2—C1	111.8 (3)	C16—C15—C20	110.8 (4)
C3—C2—H2A	109.3	C16—C15—S2	109.9 (3)
C1—C2—H2A	109.3	C20—C15—S2	110.2 (3)
C3—C2—H2B	109.3	C16—C15—H15	108.6
C1—C2—H2B	109.3	C20—C15—H15	108.6
H2A—C2—H2B	107.9	S2—C15—H15	108.6
C2—C3—C4	111.1 (4)	C15—C16—C17	111.4 (4)
C2—C3—H3A	109.4	C15—C16—H16A	109.3
C4—C3—H3A	109.4	C17—C16—H16A	109.3
C2—C3—H3B	109.4	C15—C16—H16B	109.3
C4—C3—H3B	109.4	C17—C16—H16B	109.3
H3A—C3—H3B	108.0	H16A—C16—H16B	108.0
C3—C4—C5	110.6 (4)	C18—C17—C16	110.1 (4)
C3—C4—H4A	109.5	C18—C17—H17A	109.6
C5—C4—H4A	109.5	C16—C17—H17A	109.6
C3—C4—H4B	109.5	C18—C17—H17B	109.6
C5—C4—H4B	109.5	C16—C17—H17B	109.6
H4A—C4—H4B	108.1	H17A—C17—H17B	108.1
C4—C5—C6	111.3 (3)	C19—C18—C17	110.7 (4)
C4—C5—H5A	109.4	C19—C18—H18A	109.5
C6—C5—H5A	109.4	C17—C18—H18A	109.5
C4—C5—H5B	109.4	C19—C18—H18B	109.5
C6—C5—H5B	109.4	C17—C18—H18B	109.5
H5A—C5—H5B	108.0	H18A—C18—H18B	108.1
C1—C6—C5	110.7 (3)	C20—C19—C18	111.1 (4)
C1—C6—S1	114.0 (3)	C20—C19—H19A	109.4
C5—C6—S1	105.5 (2)	C18—C19—H19A	109.4
C1—C6—H6	108.9	C20—C19—H19B	109.4
C5—C6—H6	108.9	C18—C19—H19B	109.4
S1—C6—H6	108.9	H19A—C19—H19B	108.0
C8—C7—S1	114.2 (3)	C19—C20—C15	110.8 (4)
C8—C7—H7A	108.7	C19—C20—H20A	109.5
S1—C7—H7A	108.7	C15—C20—H20A	109.5
C8—C7—H7B	108.7	C19—C20—H20B	109.5
S1—C7—H7B	108.7	C15—C20—H20B	109.5
H7A—C7—H7B	107.6	H20A—C20—H20B	108.1
C9—C8—C13	118.0 (4)		
			
O2^i^—Ag1—S1—C7	−26.26 (19)	S1—C7—C8—C13	−122.8 (4)
S2^ii^—Ag1—S1—C7	165.66 (14)	C13—C8—C9—C10	−0.1 (6)
O1—Ag1—S1—C7	65.08 (15)	C7—C8—C9—C10	176.8 (4)
O2^i^—Ag1—S1—C6	81.2 (2)	C8—C9—C10—C11	−0.2 (7)
S2^ii^—Ag1—S1—C6	−86.92 (15)	C9—C10—C11—C12	0.0 (6)
O1—Ag1—S1—C6	172.50 (16)	C9—C10—C11—C14	179.9 (4)
O2^i^—Ag1—O1—N1	−38.0 (4)	C10—C11—C12—C13	0.4 (6)
S2^ii^—Ag1—O1—N1	84.3 (3)	C14—C11—C12—C13	−179.5 (4)
S1—Ag1—O1—N1	−131.5 (3)	C9—C8—C13—C12	0.5 (6)
Ag1^iv^—O2—N1—O3	13.2 (7)	C7—C8—C13—C12	−176.4 (4)
Ag1^iv^—O2—N1—O1	−169.9 (3)	C11—C12—C13—C8	−0.7 (7)
Ag1—O1—N1—O2	142.9 (5)	C12—C11—C14—S2	110.0 (4)
Ag1—O1—N1—O3	−40.4 (6)	C10—C11—C14—S2	−69.9 (5)
C6—C1—C2—C3	−56.9 (5)	C15—S2—C14—C11	−60.3 (4)
C1—C2—C3—C4	56.7 (5)	Ag1^iii^—S2—C14—C11	−170.6 (3)
C2—C3—C4—C5	−55.6 (5)	C14—S2—C15—C16	97.4 (4)
C3—C4—C5—C6	55.9 (5)	Ag1^iii^—S2—C15—C16	−158.7 (3)
C2—C1—C6—C5	56.5 (4)	C14—S2—C15—C20	−140.2 (3)
C2—C1—C6—S1	175.2 (3)	Ag1^iii^—S2—C15—C20	−36.3 (4)
C4—C5—C6—C1	−56.9 (5)	C20—C15—C16—C17	55.8 (6)
C4—C5—C6—S1	179.4 (3)	S2—C15—C16—C17	177.9 (4)
C7—S1—C6—C1	59.5 (3)	C15—C16—C17—C18	−56.0 (6)
Ag1—S1—C6—C1	−44.0 (3)	C16—C17—C18—C19	56.5 (6)
C7—S1—C6—C5	−178.9 (3)	C17—C18—C19—C20	−57.6 (6)
Ag1—S1—C6—C5	77.6 (3)	C18—C19—C20—C15	57.1 (6)
C6—S1—C7—C8	63.2 (3)	C16—C15—C20—C19	−56.1 (6)
Ag1—S1—C7—C8	176.3 (3)	S2—C15—C20—C19	−178.0 (3)
S1—C7—C8—C9	60.3 (5)		

Symmetry codes: (i) *x*, −*y*+1/2, *z*−1/2; (ii) *x*+1, *y*, *z*; (iii) *x*−1, *y*, *z*; (iv) *x*, −*y*+1/2, *z*+1/2.

### Hydrogen-bond geometry (Å, °)

**Table:**

*D*—H···*A*	*D*—H	H···*A*	*D*···*A*	*D*—H···*A*
C14—H14A···O3^v^	0.99	2.60	3.416 (7)	140
C14—H14B···O3^iii^	0.99	2.47	3.199 (6)	130
C7—H7B···O2^i^	0.99	2.43	3.118 (6)	126

Symmetry codes: (v) *x*−1, −*y*+1/2, *z*−1/2; (iii) *x*−1, *y*, *z*; (i) *x*, −*y*+1/2, *z*−1/2.
